# Biological Characterization and Clinical Relevance of Circulating Tumor Cells: Opening the Pandora’s Box of Multiple Myeloma

**DOI:** 10.3390/cancers14061430

**Published:** 2022-03-10

**Authors:** Juan-José Garcés, Jesús San-Miguel, Bruno Paiva

**Affiliations:** Clinica Universidad de Navarra, Cancer Center Universidad de Navarra (CCUN), Centro de Investigacion Medica Aplicada (CIMA), Centro de Investigacion Biomedica en Red—Oncologia (CIBERONC), Instituto de Investigacion Sanitaria de Navarra (IDISNA), 31008 Navarra, Spain; sanmiguel@unav.es

**Keywords:** circulating tumor cell, CTC, liquid biopsy, multiple myeloma, transcriptomic characterization, genomic characterization, immunophenotype, MRD, flow cytometry, clinical trial

## Abstract

**Simple Summary:**

Bone marrow (BM) aspirates are mandatory for diagnosis and follow-up of patients with multiple myeloma (MM). However, they present two important caveats: Their invasiveness and limited scope to capture the broad tumor heterogeneity. Conversely, circulating tumor cells (CTCs) are detectable in the peripheral blood of patients with precursor and malignant disease states and have strong prognostic value. Moreover, the high genetic and transcriptomic overlap between both plasma cell compartments suggests that CTCs might reflect with notable precision the medullar clone. Furthermore, the study of CTCs could be used as a model to identify mechanisms favoring BM egression and disease spreading. Here, we summarize the state of the art on MM CTCs and provide insights on what they may offer in research and clinical scenarios.

**Abstract:**

Bone marrow (BM) aspirates are the gold standard for patient prognostication and genetic characterization in multiple myeloma (MM). However, they represent an important limitation for periodic disease monitoring because they entail an aggressive procedure. Moreover, recent findings show that a single BM aspirate is unable to reflect the complex MM heterogeneity. Recent advances in flow cytometry, microfluidics, and “omics” technologies have opened Pandora’s box of MM: The detection and isolation of circulating tumor cells (CTCs) offer a promising and minimally invasive alternative for tumor assessment and metastasis study. CTCs are detectable in premalignant and active MM states, and their enumeration has strong prognostic value, to the extent that it is challenging current stratification systems. In addition, CTCs reflect with high precision both intra- and extra-medullary disease at the phenotypic, genomic, and transcriptomic levels. Despite this high resemblance between tumor clones in distinct locations, some subtle (not random) differences might shed some light on the metastatic process. Thus, it has been suggested that a hypoxic and pro-inflammatory microenvironment could induce an arrest in proliferation forcing tumor cells to recirculate. Herein, we summarize data on the characterization of MM CTCs as well as their clinical and research potential.

## 1. Introduction

Multiple Myeloma (MM) is the second most common hematological malignancy. It is characterized by an abnormal proliferation of monoclonal plasma cells in the bone marrow (BM), which finally induces organ dysfunction known as CRAB symptoms (i.e., hypercalcemia, renal insufficiency, anemia, and bone destruction) [1]. Despite the important progress in its understanding and treatment, with remarkable advances in patient outcomes, all clinical evaluations and staging are still based on invasive BM aspirates [2]. Together with sampling and longitudinal limitations of this approach, spatial heterogeneity of tumor cells is also a significant factor that hampers a complete characterization of the disease based on single BM aspirates [3,4,5].

The presence of numerous lytic lesions throughout the axial skeleton conferred this neoplasm the surname of “multiple”, suggesting a constant dynamic spreading of tumor cells from the primary tumor to diverse BM niches through the peripheral blood (PB). Moreover, MM is preceded by well-established premalignant states, and therefore, it is an attractive model to study the metastatic process [6,7].

Liquid biopsies have emerged as a promising and minimally invasive alternative for tumor assessment. In addition to showing prognostic value [8,9,10,11,12,13,14], circulating tumor cells (CTCs) would expand the genomic view of circulating cell-free tumor DNA (ctDNA) [15,16,17,18,19] and include potential information about transcriptomic, proteomic, and metabolomic features, as well as the possibility of generating cell cultures or xenografts [20,21]. With the increasing expansion of sorting, microfluidics, and “omics” technologies [22], CTCs might revolutionize not only our comprehension of disease pathogenesis and dissemination but also patient monitorization.

Herein, we summarize different studies characterizing CTCs from a phenotypic, genetic, and transcriptomic point of view in MM, as well as their clinical applicability. Many excellent reviews expanding this topic in other cancer types are available in this journal [23,24] and elsewhere [25,26,27].

## 2. Immunophenotypic Characterization of CTCs

The presence of CTCs in MM is well known since the 90′s [28,29,30] and has been studied with different approaches (covered in this other review [23]). However, it was not until recently that a systematic comparison with its (primary) BM counterpart was performed using flow cytometry (FC) [8,31,32,33].

Since CTCs egress directly from the BM, theoretically, their phenotype should highly overlap with that of the primary BM tumor cell [8,31,32]. Indeed, the expression of classical B-cell maturation markers (e.g., CD19, CD20, CD27, CD45, or CD79) or adhesion molecules (e.g., CD44, CD54, or ITGB7) remained stable when comparing both tumor cell compartments [8,31,33].

**Figure 1:**
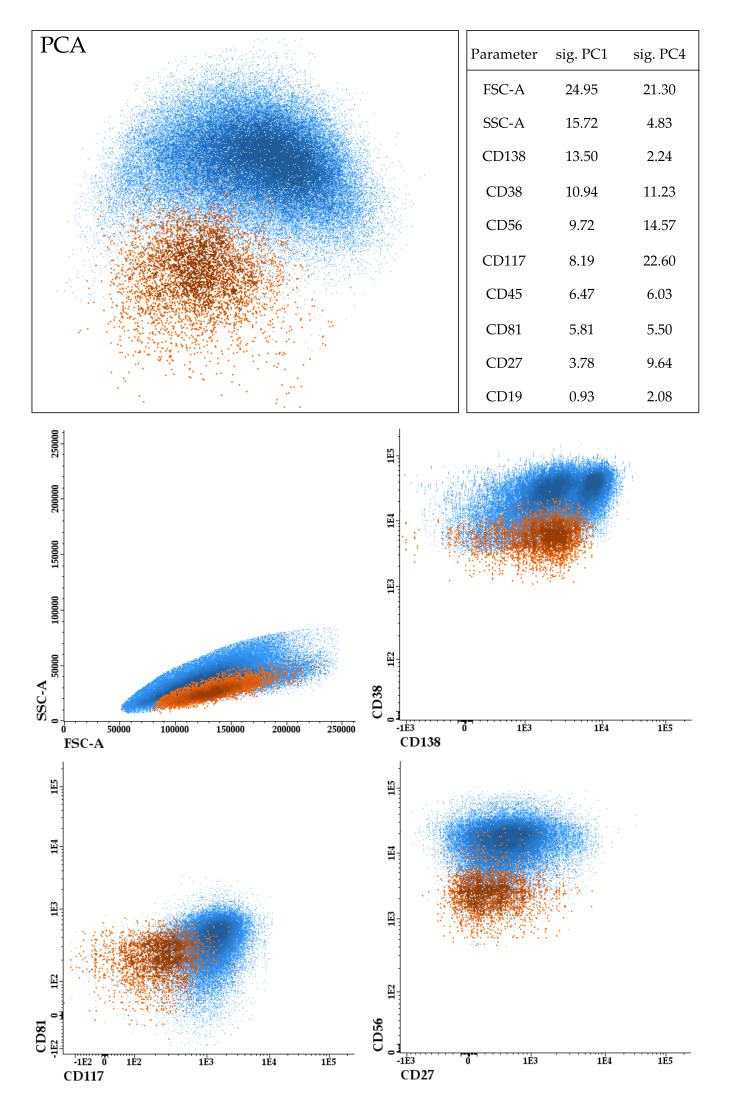
Example of the immunophenotypic features of paired bone marrow (BM) tumor cells, in green, and circulating tumor cells (CTCs), in red, from a representative patient. Results are represented by principal component analysis (PCA) based on the expression levels of eight antigens (CD19, CD27, CD38, CD45, CD56, CD81, CD117, CD138) together with forward (FSC) and side scatter (SSC). The significance (sig.) of each parameter in discriminating cells with different phenotypes is represented in the table. Of note, BM tumor cells are composed of two clones with negative and positive expression of CD138 and different morphology (FSC and SSC), whereas only CTCs without CD138 have egressed into circulation. Furthermore, CTCs show downregulation of CD38, CD56, CD81 and CD117 when compared to BM tumor cells.

However, the ability of CTCs to egress may also hide singular features favoring disease spreading through systemic circulation. Our group has shown through next-generation FC that CTCs commonly tend to cluster in unique areas of the whole space occupied by their paired BM tumor cells, suggesting commonalities and singularities in the phenotypic profiles of both plasma cells (Figure 1) [31]. Thus, the under-expression of multiple integrins, cytokine receptors, or adhesion molecules, such as CD11a, CD33, CD38, CD49e/d, CD56, CD81, CD117, or CD138, is particularly interesting to explain the presence of CTCs in the PB [8,31,33]. At the same time, each of these markers presents its own cell-intrinsic effects. For example, the reduced expression of CD81 and CD138 supports the idea that CTCs could be a more quiescent and immature population [34,35,36]. Negative expression of CD49 and CD56 has been previously associated with more invasive disease and postulated as a possible hallmark of plasma cell leukemia [37,38]. Although not directly focused on CTCs, diverse studies have suggested that chemokine receptors, such as CXCR4, CCR1, or CCR5, could be key in extra-medullary dissemination and potential targets to prevent disease progression. Interestingly, these molecules also highlight the importance of epithelial-mesenchymal transition (EMT) and hypoxia processes for tumor cell egression [39,40].

The interaction of CTCs with different immune cell types proposes new ideas for understanding tumor dissemination [41,42]. In a bright study, Szczerba et al. have shown in breast cancer that the association between neutrophils and CTCs (vs. CTCs alone) increased the expression of genes related with cell cycle progression and facilitated a faster metastasis development [43]. Although still in the beginning, platelets also displayed to have a crucial role in enhancing the endothelial adhesion and extravasation of CTCs. This platelet-based cloaking, moreover, would create a protective “shield” against external mechanical forces preserving their integrity [44]. Equally important, homotypic interactions among CTCs compose circulating clusters or microemboli already associated with prognostic effects in multiple cancers, such as lung, prostate, or breast cancer [26,45,46].

These flow-based investigational advantages present, however, some important bias: (1) Antibody panels are based on (limited) predefined knowledge, and most importantly, (2) these are manually analyzed. The advent of new methodological approaches, such as single-cell and mass and spectral cytometry, has notably expanded the number of features that can be simultaneously studied. These, in addition, are changing the paradigm of classical manual gating through more holistic analyses combining the expertise’s view with the computational power of pattern recognition [47]. Furthermore, technologies such as cell surface capture protocols could expand our restricted and predefined knowledge to a more “agnostic” and discovery-driven view of the total surfaceome (i.e., plasma membrane proteins with exposed domains towards the extracellular space), facilitating the identification of new drug targets and potential cell interactions [48].

## 3. Genetic Characterization of CTCs

Genetic characterization is a key factor for patient stratification in MM [49]. Up to now, BM aspirates have been the gold standard both for fluorescence in situ hybridization (FISH) or targeted sequencing. However, its invasiveness supposes an important caveat that restricts application only to baseline and very specific time points. Although CTCs could offer a more sustained and minimally invasive patient follow-up, their low prevalence keeps challenging their study.

To overcome this limitation, Lohr et al. used an immunodensity microfluidic device (RosetteSep™) coupled with a specific cocktail of antibodies to positively select a total of 155 single CTCs (Table 1) [50]. Other enrichment approaches were based on immunomagnetic CD138 positive selection [18]. However, next-generation FC has resulted in being the technology showing higher yields, with the ability to sort a median of 13,000 CTCs ready for downstream analyses [51,52]. Most importantly, because of the high cellular input and the multiple marker combinations, this information would be superior both in terms of quantity and, especially, quality (≥97% purity [53]).

Through targeted single-cell sequencing, Lohr et al. confirmed that CTC mutations at a single-cell level matched “bulk” BM tumor cells. Nevertheless, when comparing single CTCs with single BM tumor cells, only 7/35 (20%) mutations were shared between both tumor compartments and solely in 4/7 (57%) of patients [50]. In addition, this information would be restricted to a few preselected genes (i.e., targeted panel), which reflects an important limitation if considering the high inter- and intra-patient genetic heterogeneity in MM [5]. On the contrary, upon moving to a broader approach (i.e., whole-exome sequencing [WES]), CTCs were able to mirror up to 93% of mutations detected in BM tumor cells, including >80% of recurrently MM mutated or pan-cancer genes (e.g., *KRAS*, *BRAF*, *TP53*, etc.) [18,51,52]. The same pattern was also observed for copy number alterations, sharing classical prognostic aberrations such as amplification 1q or deletion 17p [52]. Interestingly, we replicated this concordance at the extra-medullary level finding that 84% of mutations were detectable in CTCs whenever present in extra-medullary plasmacytomas (i.e., discrete masses of tumor cells in soft tissues) [52].

According to this resemblance with BM tumor cells, CTCs could represent an ideal minimally invasive tool for risk stratification. Indeed, there are some attractive attempts that could boost their implementation into routine genetic characterization. For example, Foulk et al. coupled CTCs enumeration and isolation through the CellSearch^®^ platform with FISH analysis of main cytogenetic aberrations (i.e., del17p, t(4;14), t(14;16)) and showed 90% concordance between CTCs and BM tumor cells, both in smoldering and active MM [56]. Another promising alternative is the so-called “immuno-flowFISH”: This approach, based on imaging FC, simultaneously combines immunophenotyping with cytogenetic analysis on cells in suspension. Its application has been demonstrated by evaluating chromosome 12 deletion on chronic lymphocytic leukemia with 100% agreement with standard FISH [57].

Notwithstanding, a restricted group of mutations still persists in each cellular location. The presence of clonal mutations (i.e., cancer cell fraction [CCF] ≥ 90%) in CTCs that are subclonal (i.e., CCF < 90%) in BM tumor cells, as well as specific subclonal mutations only detectable in CTCs, reopens the question of whether this circulating population could have a distinct (or earlier) origin than primary BM tumor [51,52]. This information underpins the hypothesis that, beyond representing the main disease clone, CTCs might also consist of multiple medullar subclones from diverse BM niches [3,5], and accordingly, could reflect the disease heterogeneity with more rigor than a single BM aspirate [51,52].

## 4. Transcriptomic Characterization of CTCs

Transcriptomic profiling has been classically used to define MM subtypes with different biological features and survival rates [58]. Shedding light on mechanisms favoring cell egression might help to elucidate whether CTCs are the clone responsible for MM spreading and, eventually, disease progression [6].

Overall, the reduced number of differentially expressed genes and the highly overlapping transcriptomic profiles indicate that CTCs resemble their BM counterpart. Consequently, samples tended to cluster in a patient-specific way rather than by tumor cell source [7,50,54,55]. These findings would be additionally supported by the significant match between CTCs and BM tumor cells upon reconstructing the B-cell receptor (BCR), which points to the same clonal origin [54]. Remarkably, Lohr et al. also inferred key MM translocations, such as t(11;14) or t(6;14), from transcriptomic data and suggested a similar behavior between both tumor compartments [50].

However, it is plausible that BM egression does not constitute a random process but it is influenced by some specific differences conferring extra advantages. As we demonstrated, despite the few differentially expressed genes, these were able to segregate with notable precision CTCs from BM tumor cells and were prognostically relevant (e.g., *EMP3, LGALS1,* or *IQGAP1*) [7]. Later functional analyses indicated that CTCs were enriched in molecular hallmarks such as inflammation (e.g., interferon response, TNFα, and complement signaling), EMT, hypoxia or apoptosis (e.g., P53 pathway), and under-represented in functions related to cell cycle (e.g., DNA repair or G2M checkpoint). Under this idea, a model was proposed: The presence of hypoxic BM niches together with a pro-inflammatory microenvironment could induce an arrest in proliferation, forcing tumor cells to circulate and seek other locations to continue growing [7].

## 5. Clinical Utility of CTCs

CTCs can be identified as soon as a premalignant clonal plasma cell process is initiated. According to the EuroFlow consortium, 59% of patients with monoclonal gammopathy of undetermined significance (MGUS) have detectable CTCs as compared to 100% of cases with smoldering and overt MM [8,59]. The major difference between these entities is, however, the percentage of CTCs, with increasing (median) frequencies from 0.0002% (0.008 CTCs/µL) to 0.004% (0.16 CTCs/µL) and 0.04% (1.9 CTCs/µL) for MGUS, smoldering and active MM, respectively [8,60]. Remarkably, CTC enumeration was selected as an independent prognostic factor when comparing with classical BM assessments, both by morphology or FC [14,61].

Probably, in the clinical ground, one of the most promising applications of CTCs is their capacity for minimally invasive prognostication. In the very early disease onset (i.e., MGUS), Sanoja-Flores et al. demonstrated that the presence of ≥0.058 CTCs/µL defined a group of patients with a high risk of progression [8]. Regarding the smoldering stage, based on conventional FC (sensitivity of 10^−3^–10^−4^), the Mayo Clinic group determined that the mere detection of CTCs was an adverse prognostic factor with a median time to progression of only 10 months [13]. Upon increasing sensitivity to 10^−6^ through next-generation FC, our group has recently reported that those smoldering MM patients with ≥0.02% CTCs showed ultra-high-risk of transformation (median progression-free survival [PFS] of 11 months) [59]. In particular, early intervention of these patients with high CTC levels has been shown to substantially reduce rates of malignant transformation (PFS not reached) [59,62].

Gonsalves et al., considering active MM by using standard FC, reported that having ≥5 CTCs/µL was able to better sub-stratify the big and heterogeneous group of patients with standard-risk cytogenetics or intermediate risk according to the Revised International Staging System (R-ISS) [14,63]. The Spanish group studied with next-generation FC the prognostic impact of CTCs in uniformly treated transplant-eligible newly diagnosed MM patients (GEM2012MENOS65 clinical trial) and identified it as one the most powerful independent prognostic factor at diagnosis. They were able to define, furthermore, three risk categories: Patients with ≥0.24% CTCs (median PFS of 44 months), <0.24% CTCs (78 months), and undetectable CTCs (PFS and overall survival [OS] rates of 94% and 100%, respectively) [61]. The latter subset of patients resulted especially interesting, and here sensitivity is crucial, since it might constitute a singular group with an exceptional outcome regardless of the depth of response and other risk factors at diagnosis [8,61,64]. Despite these promising results, the standardization of these procedures is still urgently needed to implement them into routine clinical practice.

CTCs quantification could also help to redefine the concept of plasma cell leukemia, the most aggressive variant of monoclonal gammopathies and traditionally diagnosed on the presence of 20% CTCs in PB smears [65]. Thus, several groups have shown that patients with 2–5% CTCs by FC would have a similar outcome to those with plasma cell leukemias, suggesting a different strategy for managing this high-risk entity [66,67,68,69].

Finally, one of the ambitions of CTCs is to replace the BM in the evaluation of measurable residual disease (MRD) and, consequently, facilitate frequent disease monitoring in a minimally invasive way. Hence, every case with MRD positive in PB had also detectable MRD in BM, proposing that the detection of CTCs could be a surrogate of persistent MRD in BM [70]. Conversely, a remaining high rate of false negatives in PB still points out to the requirement of refining MRD assessments based on CTCs [70,71]. Interestingly, longitudinal evaluation of this circulating population highlighted that its sustained presence before and after treatment was associated with a dismal survival (median PFS around 1.5 years). Indeed, when comparing with other risk elements or depth of treatment response, the persistence of CTCs was an independent prognostic factor [70,72].

## 6. Conclusions

Legend says when Pandora opened her box, all evils escaped and spread all over the world. While ctDNA would be each individual vanished evil, CTCs represent Pandora’s box as a whole and expand possibilities for studying the medullar clone in a minimally invasive manner and understanding MM spreading.

CTCs are detectable since the very early stages of monoclonal gammopathies, and the high resemblance with their BM counterpart paves the way for explaining MM pathogenesis [6]. The ability to track specific aberrations in a minimally invasive way overcomes the limitation of serial monitoring in BM and could facilitate seeking the resistant clone and adjusting therapies over time [50,73]. At the same time, CTCs offer a potential model to interrogate metastasis in which subtle and specific features could prompt the identification of targetable mechanisms to overcome disease dissemination [6,7,74].

The box was not left totally empty when Pandora hastened to close it: At the bottom remained Hope. CTCs display a promising prognostication potential, which encourages the scientific community to revise current stratifying systems. Their enumeration, moreover, may be particularly useful in the design of new clinical trials as an alternative endpoint (e.g., absence vs. presence of CTCs, specific cutoffs, PB-based MRD) or as a new biomarker for early detection and early intervention of premalignant stages [59,75].

## Figures and Tables

**Table 1:** Genomic and transcriptomic studies comparing circulating tumor cells (CTCs) vs. bone marrow (BM) tumor cells in multiple myeloma (MM).

# Samples	#CTCs	Enrichment Technology	Sequencing Approach	Key Ideas	Ref.
7	155	RosetteSep™ + manual selection (CD138 + CD45-)	(single-cell) Targeted panel (*n* = 13)	- All mutations clinically detected by bulk BM genotyping are also detected in single CTCs. - There are mutations with greater frequency in CTCs compared to BM tumor cells. - CTCs analysis allows the detection of LOH in tumor suppressor-associated genes.	[50]
8	31,700 (median)	multiparametric FC	WES	- CTCs are detectable in 61% of MGUS and 100% of smoldering and active MM. - Around 15–20 mL of PB would suffice to sort 30,000 CTCs in a significant fraction of MM patients. - Non-recurrent but potential driver mutations and copy-number alterations are detected on CTCs.	[51]
4	-	CD138 positive selection	WES	- Tumor fraction in enriched CTCs (and ctDNA) correlates with MM progression. - Combined WES of CTCs (and ctDNA) reflects a high concordance in clonal somatic mutations (99%) and copy number alterations (81%) with BM aspirates.	[18]
8 + 10 + 35	11,090 (median)	multiparametric FC	WES + barcoded WES + CGH arrays	- Most mutations (≥82%) are simultaneously present in medullar or extra-medullar clones and can be readily monitored through the genetic characterization of CTCs (both by WES and CGH arrays). - Other abnormalities with prognostic value (e.g., amp1q, or *TP53*) or potential role in progression but not routinely tested (e.g., *MYC*) are detectable in CTCs whenever present in BM tumor cells.	[52]
2	21	RosetteSep™ + manual selection (CD138 + CD45-)	scRNAseq (Smart-seq2)	- Transcriptomic profiling of CTCs reproduces gene expression of BM tumor cells and can be used to detect targetable antigens (e.g., CD38, SLAMF7, and BCMA). - CTCs are feasible for inferring single-cell expression for translocation-based classification.	[50]
29 + 3	5200 (median) + 266	multiparametric FC	Expression arrays + scRNAseq (Precise WTA Single Cell Assay, BD)	- There is a significant correlation in gene expression between paired CTCs and BM tumor cells (both at single-cell and bulk levels). - Subtle differences between CTCs and BM tumor cells display prognostic potential. - It is suggested that a hypoxic and pro-inflammatory BM microenvironment induces an arrest in proliferation, forcing tumor cells to circulate.	[7]
15	2299 (median)	FACS (CD138+ CD38+) (in plate)	scRNAseq (MARS-seq)	- CTC signatures highly resemble the BM transcriptional state(s), with few changes likely resulting from the different environments. - The tumor load in the BM and the PB differs by several orders of magnitude.	[54]
5	44,779 (median)	-	scRNAseq	- The absence of specific clusters and the transcriptional similarity suggest that CTC levels are not driven by a transcriptionally-primed migratory clone. - BM tumor cell proliferation is a significant differential factor between high and low levels of CTCs.	[55]

Number of samples refers only to patient-matched CTCs and BM tumor cells used for paired comparisons. Number of CTCs is given as absolute values if nothing different is indicated. FC, flow cytometry; FACS, fluorescence-activated cell sorting; WES, whole-exome sequencing; CGH, comparative genomic hybridization (array); scRNAseq, single-cell RNA-sequencing; BD, Becton Dickinson; MGUS, monoclonal gammopathy of undetermined significance; PB, peripheral blood; LOH, loss of heterozygosity; ctDNA, circulating-free tumor DNA.
